# Transcriptome Analyses of a Salt-Tolerant Cytokinin-Deficient Mutant Reveal Differential Regulation of Salt Stress Response by Cytokinin Deficiency

**DOI:** 10.1371/journal.pone.0032124

**Published:** 2012-02-15

**Authors:** Rie Nishiyama, Dung Tien Le, Yasuko Watanabe, Akihiro Matsui, Maho Tanaka, Motoaki Seki, Kazuko Yamaguchi-Shinozaki, Kazuo Shinozaki, Lam-Son Phan Tran

**Affiliations:** 1 Signaling Pathway Research Unit, RIKEN Plant Science Center, Yokohama, Kanagawa, Japan; 2 Agricultural Genetics Institute, Vietnamese Academy of Agricultural Science, Hanoi, Vietnam; 3 Plant Genomic Network Research Team, RIKEN Plant Science Center, Yokohama, Kanagawa, Japan; 4 Japan International Research Center for Agricultural Sciences, Tsukuba, Ibaraki, Japan; 5 Gene Discovery Research Group, RIKEN Plant Science Center, Yokohama, Kanagawa, Japan; Instituto de Biología Molecular y Celular de Plantas, Spain

## Abstract

Soil destruction by abiotic environmental conditions, such as high salinity, has resulted in dramatic losses of arable land, giving rise to the need of studying mechanisms of plant adaptation to salt stress aimed at creating salt-tolerant plants. Recently, it has been reported that cytokinins (CKs) regulate plant environmental stress responses through two-component systems. A decrease in endogenous CK levels could enhance salt and drought stress tolerance. Here, we have investigated the global transcriptional change caused by a reduction in endogenous CK content under both normal and salt stress conditions. Ten-day-old *Arabidopsis thaliana* wild-type (WT) and CK-deficient *ipt1,3,5,7* plants were transferred to agar plates containing either 0 mM (control) or 200 mM NaCl and maintained at normal growth conditions for 24 h. Our experimental design allowed us to compare transcriptome changes under four conditions: WT-200 mM vs. WT-0 mM, *ipt1,3,5,7*-0 mM vs. WT-0 mM, *ipt1,3,5,7*-200 mM vs. *ipt1,3,5,7*-0 mM and *ipt1,3,5,7*-200 mM vs. WT-200 mM NaCl. Our results indicated that the expression of more than 10% of all of the annotated *Arabidopsis* genes was altered by CK deficiency under either normal or salt stress conditions when compared to WT. We found that upregulated expression of many genes encoding either regulatory proteins, such as NAC, DREB and ZFHD transcription factors and the calcium sensor SOS3, or functional proteins, such as late embryogenesis-abundant proteins, xyloglucan endo-transglycosylases, glycosyltransferases, glycoside hydrolases, defensins and glyoxalase I family proteins, may contribute to improved salt tolerance of CK-deficient plants. We also demonstrated that the downregulation of photosynthesis-related genes and the upregulation of several *NAC* genes may cause the altered morphological phenotype of CK-deficient plants. This study highlights the impact of CK regulation on the well-known stress-responsive signaling pathways, which regulate plant adaptation to high salinity as well as other environmental stresses.

## Introduction

Salt stress is one of the major abiotic stresses experienced by plants worldwide, affecting approximately 7% of the world's total land area [1], [2]. Mild salt stress primarily affects plant development, agronomy traits and agricultural productivity, but extremely high salinity stress can lead to plant death. In addition, climate change and declining water quality are of great concern because they contribute to land degradation by causing high salinity levels in soil. Thus, salt stress has been considered an increasingly serious problem underscoring the importance of developing salt-tolerant plants, through the use of genetic engineering, which are capable of surviving under saline conditions [2]–[5]. Salt stress negatively impacts photosynthesis, energy production, lipid metabolism, nutrient acquisition, the integrity of cellular membranes and the activity of various enzymes, thereby leading to a number of destructive processes, such as water deficit, hyperosmotic stress, secondary oxidative stress, homeostasis disruption and ionic toxicity [6]–[9].

To cope with salinity stress, plants employs various mechanisms, at both the whole plant and cellular levels, which are controlled by a variety of genes and signaling pathways and are expressed and activated at different times during the life of a plant [4], [6], [7]. With the availability of genomic sequences from various plant species and recent advances in microarray technologies, genes associated with high salinity tolerance have been identified on a large scale at a genome-wide level [2], [10], [11]. Together with other omic technologies, such as proteomics and metabolomics, transcriptomics has contributed significantly to the elucidation of stress responses [12]–[14].

Cytokinins (CKs) are master regulators of plant growth and development, and were recently shown to control plant adaptation to salt stress [15]–[19]. The most common naturally occurring isoprenoid CKs are *N* ^6^-isopentenyladenine (iP), *trans*-zeatin (*t*Z) and *cis*-zeatin (*c*Z). Experimental evidence has demonstrated that in many plant species, including *Arabidopsis thaliana*, *t*Z is more abundant and more active than *c*Z, whereas in some other species, such as maize (*Zea mays*), *c*Z is an abundant and bioactive CK [20]. In *Arabidopsis*, CK biosynthesis is controlled by nine adenosine phosphate-isopentenyltransferases (IPTs), which are divided into two classes of IPTs: the ATP/ADP IPT class (IPT1, IPT3, IPT4, IPT5, IPT6, IPT7 and IPT8), which are responsible for biosynthesis of iP and *t*Z CKs, and the transfer RNA IPT class (IPT2 and IPT9), which are responsible for biosynthesis of *c*Z CKs [21]. Loss-of-function studies using various combinations of *Arabidopsis ipt* mutants indicated that disruption of *IPT1*, *IPT3*, *IPT5* and *IPT7* resulted in significant reductions in CK levels in *Arabidopsis* seedlings. The *ipt1,3,5,7* quadruple mutant, at 18-day-old, was reported to contain less than 20% of iP riboside 5′-phosphates, iP riboside, *t*Z riboside 5′-phosphates, *t*Z riboside, *t*Z-7-N-glucoside, *t*Z-9-N-glucoside and *t*Z-O-glucoside and less than 50% of the free-base iP and *t*Z in comparison with the WT [21]. Recently, analysis of the CK-deficient *ipt1,3,5,7* mutant under salt stress conditions demonstrated that this mutant possesses a strong salt-tolerant phenotype, suggesting the importance of CKs in regulating salt tolerance.

To identify genes involved in salt stress tolerance and regulated by CKs, as well as to elucidate molecular mechanisms associated with CK-regulated salt tolerance, we have conducted a genome-wide transcriptional analysis to compare the transcriptomes of the wild-type (WT) and the CK-deficient *ipt1,3,5,7* mutant under both normal and salt stress conditions using whole genome *Arabidopsis* microarrays. Our results suggest that reduction of bioactive CK levels induced changes in gene expression of many regulatory and functional genes, including transcription factors (TFs), calcium sensors, high affinity K^+^/Na^+^ co-transporter proteins, heat shock proteins, late embryogenesis-abundant (LEA) proteins, xyloglucan endo-transglycosylases (XTR), glycosyltransferases (GTs), glycoside hydrolases (GHs), defensins and glyoxalase I family proteins, leading to improved salt and drought stress tolerance of the *ipt1,3,5,7* mutant.

## Materials and Methods

### Plant materials and salt stress treatment

WT *Arabidopsis* (Columbia ecotype) and CK-deficient *ipt1,3,5,7* (Columbia ecotype) seeds [21] were sowed on germination medium (GM) agar plates supplemented with 3% sucrose and kept for 4 days at 4°C. Plates were then transferred to 22°C and incubated for 10 days (22°C, 16 h light/8 h dark cycle, 60 µmol m^−2^ s^−1^ photon flux density). For microarray analyses, the 10-day-old plants grown on GM media were transferred onto 0.5× MS plates without sucrose, containing either 0 mM (control) or 200 mM NaCl and maintained for a period of 24 h. Three independent experiments were performed for each condition. The samples were collected as three biological replicates (10 plants/replicate), frozen in liquid nitrogen and stored at −80°C until used for RNA extraction.

### RNA purification and microarray analyses

Total RNA was extracted with TRIZOL Reagent according to the supplier's instructions (Invitrogen, Carlsbad, CA, USA). The microarray analyses, hybridization, data analyses and data mining were carried out as previously described [22]. Briefly, cDNAs were synthesized using 500 ng of total RNA and labeled with one color (Cy3) using a Quick Amp labeling kit (Agilent Technologies), followed by fragmentation and hybridization to the *Arabidopsis* Oligo 44K DNA microarray (Ver. 4.0, Agilent Technologies). Three biological replicates were performed for each treatment, making a total of 12 hybridizations. All arrays were scanned with a microarray scanner (G2505B, Agilent Technologies) and analyzed using GeneSpring Ver. 11 (Agilent Technologies). The Student's *t*-test (p-value) was used as a parametric test and the Benjamini and Hochberg False Discovery Rate (q-value) procedure was used to control the certainty level. Genes with at least a 2-fold difference in their expression levels and a q-value<0.05 were considered to be differentially expressed. The raw microarray data and the detailed protocol were deposited in the Gene Expression Omnibus database (http://www.ncbi.nlm.nih.gov/geo/browse/?view=series) with GEO ID: GSE32087.

### qRT-PCR

RNA samples from three biological replicates were used in the qRT-PCR validation of the microarray data. cDNA synthesis and qRT-PCR were performed according to previously described methods [23], [24]. The primer pairs that were used in qRT-PCR reactions are listed in Table S1. *UBQ10* was used as an internal control for expression analyses.

### Search for stress-inducible *cis*-motifs in promoter regions of upregulated genes

The 1-kb promoter regions upstream of transcriptional start sites (+1) were retrieved from the *Arabidopsis* TAIR10 (ftp://ftp.arabidopsis.org/home/tair/Sequences/blast_datasets/TAIR10_blastsets/upstream_sequences/). The existence of 12 stress-inducible *cis*-elements [25]–[27] was searched in the 1-kb promoter region of each upregulated gene as previously described [28].

### Determination of biological process categories of the upregulated and downregulated gene sets using gene ontology

The biological process categories of the up- and downregulated genes identified in four comparisons were determined as previously described [29], [30]. The gene ontology (GO) terms assigned to each of the upregulated or downregulated genes were counted using the data set of *Arabidopsis* TAIR10 (ftp://ftp.arabidopsis.org/home/tair/Ontologies/Gene_Ontology/). The top 20 most abundant GO terms were subsequently used to classify the genes into biological process categories.

### MapMan analysis

The list containing differentially expressed genes obtained from four comparisons was annotated and categorized into functional groups using MapMan version 3.5.1 (http://mapman.gabipd.org/web/guest/mapman) according to the standard protocol [31], [32].

## Results and Discussion

### Expression profiling of WT and CK-deficient *ipt1,3,5,7* plants using microarrays under normal and salt stress conditions

We have previously reported that the levels of free-base iP and *t*Z as well as their riboside and ribotide forms in 10-day-old *Arabidopsis ipt1,3,5,7* seedlings were reduced to less than 25% of the WT levels [16]. Furthermore, using the system that was developed for salt-tolerance studies, we have shown that the 10-d-old *ipt1,3,5,7* plants displayed a stronger salt-tolerant phenotype than WT [16], [18]. Thus, to investigate the molecular changes brought about by CK deficiency and that contribute to enhanced salt stress tolerance, the same system was used for salt treatment. Ten-day-old WT and CK-deficient *ipt1,3,5,7* plants were transferred onto plates containing 0 mM and 200 mM NaCl for 24 h. The collected samples were then subjected to microarray analyses using an *Arabidopsis* Oligo 44K DNA microarray (Ver. 4.0) for profiling the transcripts of WT and *ipt1,3,5,7* plants in the absence and presence of 200 mM NaCl. The results of the microarray analyses are available in Table S2 or can be accessed through Gene Expression Omnibus (accession number: GSE32087).

**Figure 1:**
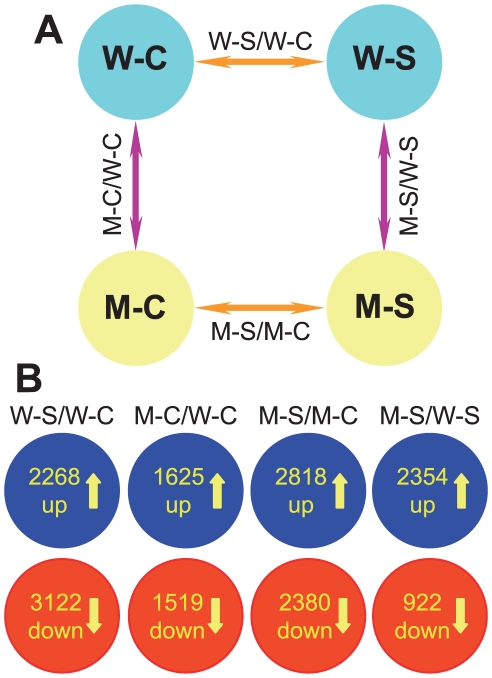
Diagrams showing experimental design, comparisons and compilation of genes with altered expression in each comparison. (A) Diagrams showing experimental design and comparisons. (B) Diagrams showing compilation of genes with altered expression in each comparison. W-S, WT-200 mM NaCl; W-C, WT-0 mM NaCl; M-S, *ipt1,3,5,7*-200 mM NaCl; M-C, *ipt1,3,5,7*-0 mM NaCl; W-S/W-C, WT-200 mM NaCl vs. WT-0 mM NaCl; M-C/W-C, *ipt1,3,5,7*-0 mM NaCl vs. WT-0 mM NaCl; M-S/M-C, *ipt1,3,5,7*-200 mM NaCl vs. *ipt1,3,5,7*-0 mM NaCl; M-S/W-S, *ipt1,3,5,7*-200 mM NaCl vs. WT-200 mM NaCl.

**Figure 2:**
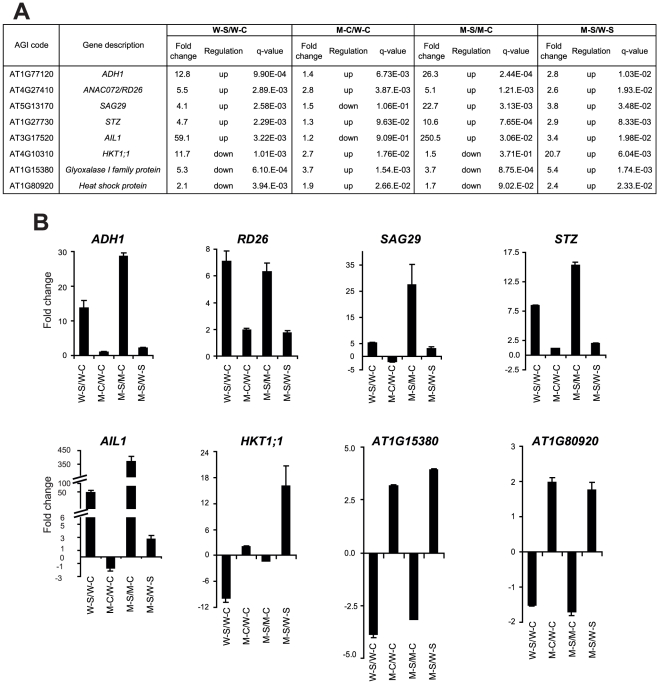
Confirmation of microarray data by qRT-PCR analysis. Eight genes were selected and their expression profiles were assessed by qRT-PCR in all four plant samples to verify the microarray data. (A) Fold changes were obtained from microarray analysis. (B) Fold changes were obtained from qRT-PCR analysis. Fold changes were calculated from the expression data obtained by qRT-PCR in all four samples. Relative quantitation of expression was calculated using 2^−ΔCt^ method and *UBQ10* as endogenous control. cDNAs were obtained from three biological replicates.

This experimental design enabled us to investigate and compare the differentially expressed transcriptomes of WT and CK-deficient *ipt1,3,5,7* plants in four ways: WT-200 mM vs. WT-0 mM (comparison W-S/W-C), *ipt1,3,5,7*-0 mM vs. WT-0 mM (comparison M-C/W-C), *ipt1,3,5,7*-200 mM vs. *ipt1,3,5,7*-0 mM (comparison M-S/M-C), *ipt1,3,5,7*-200 mM vs. WT-200 mM NaCl (comparison M-S/W-S) (Figure 1A). In comparisons W-S/W-C and M-S/M-C, we sought to identify, in a global and unbiased manner, genes whose expression was modulated in response to salt stress in WT and CK-deficient plants. Comparisons M-C/W-C and M-S/W-S allowed us to detect the changes in gene expression induced by CK deficiency under normal and salt stress conditions, respectively. Using the criteria of the ratio change > = 2 and q-value<0.05, 2268, 1625, 2818 and 2354 genes were found to be upregulated and 3122, 1519, 2380 and 922 genes were downregulated in comparisons W-S/W-C, M-C/W-C, M-S/M-C and M-S/W-S, respectively (Figure 1B, Tables S3, S4, S5, S6). Eight genes were selected to validate our microarray data by qRT-PCR. The results shown in Figure 2 supported the reliability of the microarray data.

**Figure 3:**
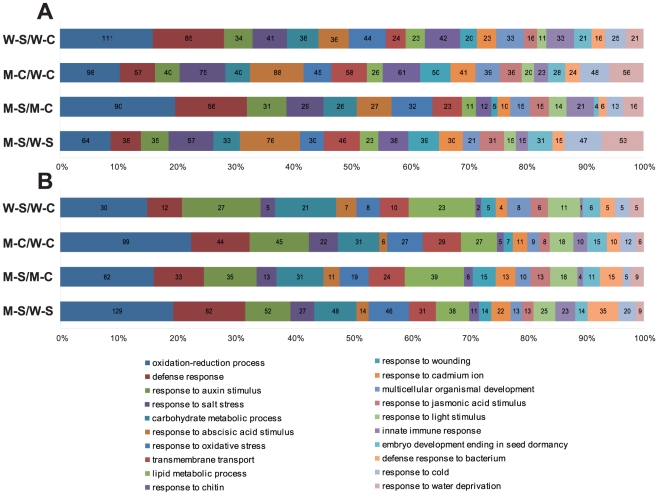
Distributions of upregulated (A) and downregulated (B) gene sets identified in four comparisons into the biological process categories based on GO analysis. The top 20 most abundant biological process categories are shown. Gene numbers are displayed for each category.

**Figure 4:**
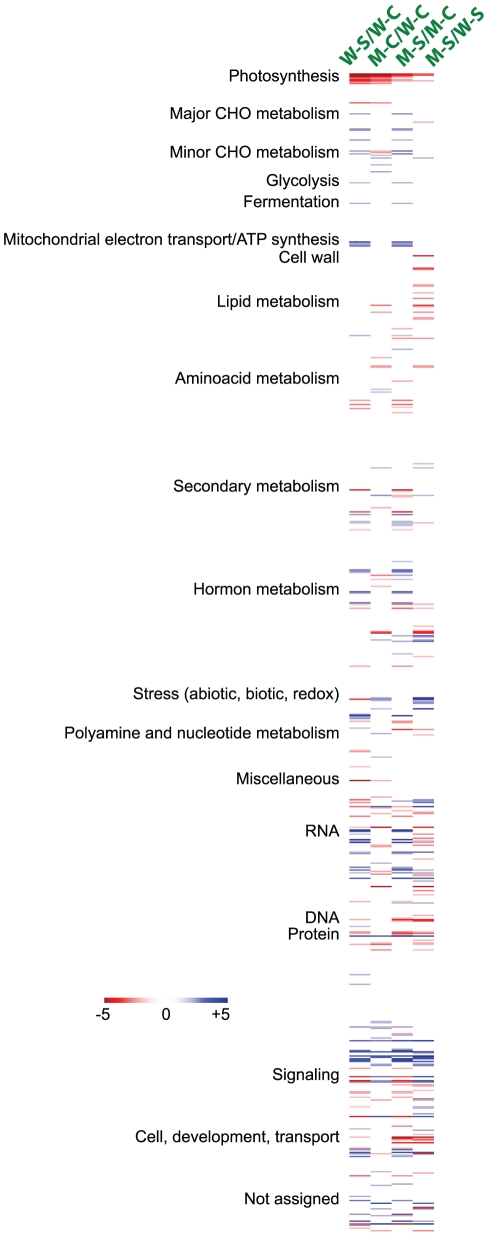
MapMan analysis showing molecular functional categories of genes with altered expression in four comparisons. Changes in expression levels are indicated by color scales with saturation at five-fold changes. Blue and red color gradients indicate an increase or decrease in transcript abundance, respectively.

All four differentially expressed gene sets were subsequently classified into biological process categories based on GO analysis. Figure 3 illustrated the distribution of the up- and downregulated gene sets in the 20 most abundant GO terms among which many abiotic stress-related categories, such as “response to salt stress”, “response to water deprivation”, “response to ABA”, “response to oxidative stress” and “response to cold” were found. A significantly higher proportion of salt stress-responsive genes were detected in each of four upregulated gene sets (Figure 3A) in comparison with the respective downregulated gene sets (Figure 3B). Additionally, genes responsive to ABA and water stress were also found in higher abundance in all four upregulated gene sets than in the corresponding downregulated ones, which is consistent with the strong relationship observed among ABA, water stress and high salinity [33], [34]. Moreover, we found that more stress-related genes, such as those classified into “response to salt stress”, “response to water deprivation”, “response to ABA” and “response to cold”, are upregulated in comparison M-C/W-C or in comparison M-S/W-S than in comparison W-S/W-C (Figure 3A); a phenomenon that may enable the CK-deficient plants to cope better with stresses as previously reported [16], [35]. MapMan was also used to visualize the gene expression data to understand relations in terms of molecular functional categories among the four comparisons and find possible molecular mechanism(s) connecting salt stress response and CK deficiency at the genomic level as shown in Figure 4 and discussed in details in appropriate sections.

In addition, stress-responsive *cis*-elements have been reported for their essential roles in determining the stress-responsive expression patterns of genes, facilitating the stress-related functional classification [28]–[30]. Over the years, extensive promoter analyses have identified 12 stress-inducible *cis*-elements, which are important molecular switches involved in the transcriptional regulation of a dynamic network of gene activities controlling abiotic stress responses [25]–[27]. Therefore, to grasp the overall representation of stress-related genes among the upregulated genes identified in four comparisons we searched for the existence of these *cis*-elements in the 1-kb promoter region of each of upregulated genes identified in four comparisons. Data shown in Tables S3A, S4, S5, S6A indicate that a significant portion of upregulated gene sets are stress-responsive. It should be noticed that all 12 *cis*-elements used were identified as either drought-, cold- and/or ABA-inducible elements [25]–[27]. However, the results of the *cis*-element analysis are still useful because significant overlap has been reported for the gene sets upregulated by drought-, high-salinity-, cold- and ABA-inducible genes [33], [34]. The percentages of stress-inducible genes might be higher if specific salt stress-inducible *cis*-motifs were also be included into the *in silico* analysis (Tables S3A, S4, S5, S6A). Unfortunately, the identity of salt stress-inducible *cis*-motifs is currently unknown.

### Identification of salt stress-responsive genes in WT plants treated by the “plate method”

Several groups have reported genome-wide expression analyses of salt stress response genes in *Arabidopsis* [33], [36], [37]. However, in these reports the methods used to treat the plants were different from ours. Plate-grown young seedlings were either transferred to a high saline solution (200–250 mM) and grown hydroponically for a short period of time (1–12 h) (solution method) [33], [37], or soil-grown plants were irrigated with saline solution (150 mM) within 24 h (irrigation method) to generate salt-treated samples for investigation [36]. In another independent study, Krishnaswamy et al. (2008) allowed *Arabidopsis* seeds to germinate and grow on 0.5× MS media containing 100 mM NaCl for 14 days (long-term treatment with mild concentration of NaCl). Here, we used the “plate method”, which was developed for studying salt stress tolerance. This method, therefore, enabled us to identify molecular changes that were directly linked to a salt-tolerance phenotype. From the experiment comparing expression profiles of WT with and without 200 mM NaCl treatment for 24 h (comparison WS/WC), we found that expression of 2268 and 3122 genes were increased and decreased, respectively, at least two-fold (Figure 1B, Tables S3A and S3B). Although the treatment methods were different, the salt-inducible genes identified by our study significantly overlapped with those found by other groups [33], [36]–[38], indicating that all of these methods (soil-grown plants, plate-grown plants, treatment in solution or growth on agar plates containing salt) and different treatment time periods (shock short-term or long-term treatments) elicit similar responses at the transcriptional level. For instance, many high salinity-responsive genes known from the literature, such as several AP2-EREBP-type *DREB*s, *RAP2*, *NAC*s, *NCED2*, *NCED3*, *RD20*, *RD22*, *RD29B*, *PP2C* and diverse *LEA* genes, were strongly induced (Tables S3A) [33], [36]–[38].

The differentially expressed genes could be classified into regulatory and functional categories. For the regulatory category, many upregulated genes were grouped into transcription factor (TF), protein degrading and protein modification groups. In the TF group, the AP2-EREBPs, such as DREB1A, DREB2A and DREB2B, zinc finger-type, MYB, WRKY and NAC TF encoding genes were present in high numbers. Heat shock TF and heat shock protein encoding genes were also found among the genes with increased transcript abundance. For the protein modification groups, we identified many salt-inducible genes encoding kinases, such as CIPKs and MAPKKKs, and PP2C proteins. The functional category contained many salt-regulated genes encoding LEA proteins, ABA metabolism-related proteins, osmoprotectant biosynthesis-related proteins, transporters and detoxification enzymes which were upregulated (Table S3A). These types of proteins are known to enable plants to adapt better to salt stress [6], [39]. As for genes that were downregulated by salt stress, many photosynthesis-related genes were found (Table S3B). This finding is consistent with previously published results [33], [36]–[38].

### CK deficiency alters expression of a subset of stress-responsive genes under normal conditions

**Figure 5:**
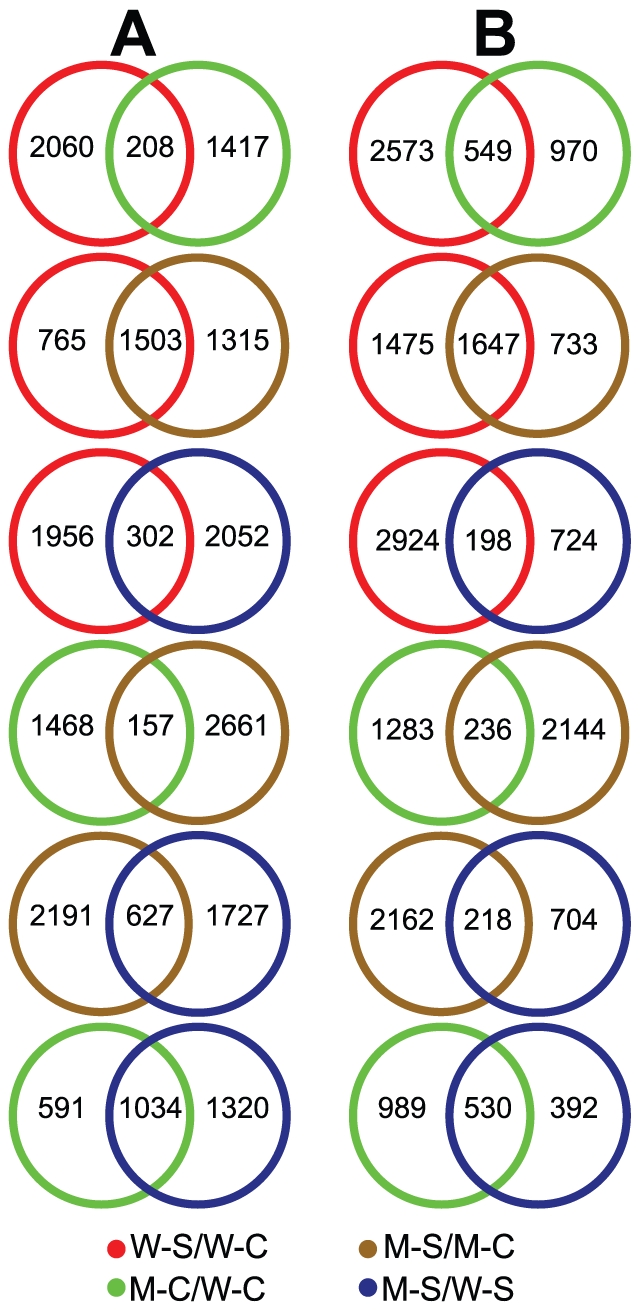
Venn diagram analysis showing the overlapping and non-overlapping upregulated (A) and downregulated (B) gene sets between each pair of comparisons.

Next, because CK-deficiency enhanced the tolerance of the CK-deficient *ipt1,3,5,7* mutant to high salinity compared with the WT [16], we examined differences in gene expression between the *ipt1,3,5,7* mutant and WT plants in the absence of NaCl in the M-C/W-C comparison. We identified a total of 3144 genes that responded to CK deficiency, representing more than 10% of the total number of genes in the *Arabidopsis* genome, of which 1625 genes were upregulated and 1519 genes were downregulated (Figure 1B, Tables S4A and S4B). Both the down- and upregulated gene sets identified in comparisons W-S/W-C and M-C/W-C were subjected to Venn diagram analysis to identify overlapping genes between the two comparisons. As shown in Figure 5, many genes that were upregulated or downregulated in the untreated *ipt1,3,5,7* mutant were also upregulated or downregulated, respectively, in the salt-treated WT, indicating significant overlap of gene expression profiles between the untreated *ipt1,3,5,7* mutant and the salt-treated WT plants (comparisons W-S/W-C and M-C/W-C). These data might explain the better adaptation of the CK-deficient plants to salt stress. In addition, expression of the *IPT1*, *3*, *5* and *7* genes was significantly decreased in the *ipt1,3,5,7* mutant background, further demonstrating the reliability of our microarray analyses (Table S4B).

A number of genes encoding regulatory proteins, such as AP2-EREBP-, bZIP-, NAC-, WRKY- and ZFHD-type TFs, including the well-known DREB2A, ANAC021/NAC1, ANAC092/AtNAC2, ANAC055/AtNAC3, ANAC072/RD26 and ZFHD1 TFs, and protein kinases, including CIPKs and CDPKs, were upregulated in the *ipt1,3,5,7* mutant (Table S4A). The *ANAC092*, *ANAC055*, *ANAC072* and *ZFHD1* genes were induced by ABA and salt stress, but *DREB2A* has been shown to be induced by salt stress and not by ABA [40]–[43], suggesting that both the ABA-dependent and ABA-independent TFs are regulated by CKs. It was reported that overexpression of *ANAC021* and *ANAC092* in *Arabidopsis* promoted lateral root development, which is in agreement with enhanced lateral root development observed in the *ipt1,3,5,7* mutant [21], [41], [44]. On the other hand, *Arabidopsis* transgenic plants overexpressing the active form of *DREB2A* exhibited enhanced salt tolerance [45], whereas those overexpressing either *ANAC055*, *ANAC072* or *ZFHD1* enhanced drought tolerance [40], [42]. However, there is no information regarding the salt-responsive phenotype of the *ANAC055*, *ANAC072* or *ZFHD1* overexpressing plants. In good accordance with these reports, the CK-deficient *ipt1,3,5,7* mutant also exhibited improved drought tolerance [16].

Many genes encoding functional proteins, such as glyoxalase I proteins, XTRs, GTs, GHs and defensins (PDF1.2), were found among the upregulated genes in the *ipt1,3,5,7* mutant in comparison with WT. As for glyoxalase I proteins, overexpression of *Brassica juncea* glyoxalase I and *Oryza sativa* glyoxalase II encoding genes, either alone or together, conferred improved salinity tolerance to tobacco (*Nicotiana tabacum*) [46]. In *Arabidopsis*, glyoxalase I proteins encoding genes were reported as potential downstream targets of drought and salt stress-responsive NAC TFs, such as ANAC019, ANAC055 and ANAC072 [40], [47]. It was suggested that glyoxalase enzymes are involved in the glutathione-based detoxification of methylglyoxal, a toxic aldehyde formed primarily as a byproduct of carbohydrate and lipid metabolism, thereby enhancing salt stress tolerance [48], [49]. XTR, GT and GH enzymes are known to play important roles in the modification of the xyloglucan cross-links that control the extensibility and strength of the plant cell wall [50]–[52]. Defensins, in addition to being implicated in mediating plant responses to pathogens, are also believed to play a role in salt stress tolerance because their transcript abundance is much higher in the *Arabidopsis* relative halophyte salt cress (*Thellungiella halophila*) than in *Arabidopsis* [53]. Moreover, induced expression of the defensin *PDF1.2* gene was reported to result in enhanced root development, consistent with the better enhanced root development observed with the *ipt1,3,5,7* mutant [54].

It has been established that CKs also affect plant disease resistance [55]. In the CK-deficient mutant we detected 35 and 10 disease resistance-related genes with increased and diminished expression levels, respectively, as compared with WT (Tables S4A and S4B). Almost all of the up- and downregulated disease resistance-related genes encode CC-NBS-LRR or TIR class proteins, which mediate resistance to various plant pathogens [56]–[58]. Many NBS-LRR-encoding genes are upregulated in response to pathogen attack [56]. Therefore, it would be interesting to examine the relationship between the functions of CKs, CK signaling and NBS-LRR proteins in pathogen responses. Furthermore, among the genes downregulated by CK deficiency, MapMan analysis has identified many photosynthesis related genes (Figure 4, Table S4B). Consistent with our data, genes related to photosynthesis were found to be upregulated in *Arabidopsis* plants treated with CKs [59]. All together, these results suggest that reduced photosynthesis may cause the retardation of shoot growth observed with CK-deficient plants which is referred to as CK deficiency syndrome [21], [60]. The restraint of shoot growth has been regarded as an advantageous growth adjustment in response to environmental cues, including salt stress, and is known to be an important mechanism of plant survival [61]–[63].

### Modulation of salt stress-responsive genes in the CK-deficient *ipt1,3,5,7* mutant

To further investigate the effect of both salt stress and CK deficiency on gene expression, we carried out comparison M-S/M-C. A total of 2818 and 2380 genes with increased and decreased expression levels, respectively, were identified from this comparison (Figure 1B, Tables S5A and S5B). The altered gene expression profile of the salt-treated *ipt1,3,5,7* mutant significantly overlapped with that of the salt-treated WT. Specifically, when the up- and downregulated gene sets identified in comparison M-S/M-C were compared with the corresponding gene sets from comparison W-S/W-C, overlap was observed for 53.34% and 69.2% of the up- and downregulated gene sets of comparison M-S/M-C, respectively, indicating that expression of many salt stress-responsive genes was altered in comparison M-S/M-C as expected (Figure 5, comparisons W-S/W-C and M-S/M-C). However, many overlapping genes exhibited differential expression levels in the degree of modulation of transcript abundance. Among six DREB-type TF encoding genes, expression of *DREB1A*, *DREB1B*, *DREB1C*, *DREB1D* and *DREB2B* was increased by 3.7-, 60.6-, 14-, 31.8- and 18.5-fold, respectively, in salt-treated WT seedlings, but by 7.1-, 122.4-, 22.1-, 89- and 24.6-fold in salt-treated *ipt1,3,5,7* seedlings compared with respective untreated controls (Table 1, comparisons M-S/M-C and W-S/W-C). Similarly, among 17 overlapping salt-inducible *NAC* genes, the majority of them (11 genes), including *ANAC013*, *ANAC019*, *ANAC036*, *ANAC044*, *ANAC055*, *ANAC071*, *ANAC079*, *ANAC081*, *ANAC085*, *ANAC090* and *ANAC102* were more highly induced in the *ipt1,3,5,7* mutant than in the WT under salt stress. On the other hand, only two genes, *ANAC061* and *ANAC072*, exhibited smaller inductions in comparison M-S/M-C than in comparison W-S/W-C. All of the overlapping upregulated ABA-responsive LEA protein encoding genes also showed higher increases in transcript abundance in salt-treated *ipt1,3,5,7* than in salt-treated WT compared with respective untreated controls (Table 1, comparisons M-S/M-C and W-S/W-C). It should be noted that CK deficiency does not have a significant effect on the expression of the *DREB*, *NAC* and *LEA* genes, except for *ANAC055*, *ANAC072*, *ANAC079* and *DREB2A* (Table 1, comparison M-C/W-C), suggesting that transcript abundance of these genes was altered in salt-treated *ipt1,3,5,7* plants in a salt-dependent rather than CK-dependent manner.

**Table 1:** Gene upregulated in comparisons W-S/W-C and M-S/M-C in comparison with comparison M-C/W-C.

		W-S/W-C			M-C/W-C			M-S/M-C		
AGI	Description	Fold change	Regulation	q-value	Fold change	Regulation	q-value	Fold change	Regulation	q-value
*AT1G01010*	*ANAC001*	2.14	up	9.5E-03	1.37	up	4.6E-02	2.17	up	9.8E-04
*AT1G01720*	*ANAC002*	4.10	up	8.6E-04	1.18	up	7.8E-03	3.91	up	9.2E-04
*AT1G32870*	*ANAC013*	2.50	up	1.1E-03	1.10	down	1.4E-01	5.65	up	4.3E-04
*AT1G52890*	*ANAC019*	8.18	up	1.7E-02	1.37	up	1.3E-01	19.15	up	8.7E-04
*AT1G77450*	*ANAC032*	3.25	up	1.4E-03	1.39	up	5.6E-03	3.00	up	5.4E-04
*AT2G17040*	*ANAC036*	3.44	up	7.0E-03	1.13	up	7.4E-01	4.43	up	1.7E-02
*AT3G01600*	*ANAC044*	2.70	up	2.1E-02	1.33	up	3.9E-01	5.23	up	2.3E-03
*AT3G15500*	*ANAC055*	7.40	up	1.9E-03	2.28	up	2.1E-02	9.95	up	1.3E-03
*AT3G44350*	*ANAC061*	6.78	up	3.9E-02	4.84	up	6.5E-02	3.40	up	5.0E-03
*AT4G01550*	*ANAC069*	2.22	up	7.3E-03	1.16	up	3.8E-01	2.34	up	3.6E-03
*AT4G17980*	*ANAC071*	2.63	up	1.1E-03	1.10	up	2.4E-01	3.43	up	7.4E-04
*AT4G27410*	*ANAC072*	5.51	up	2.9E-03	2.83	up	3.9E-03	5.10	up	1.2E-03
*AT5G07680*	*ANAC079*	2.30	up	1.3E-03	3.54	up	1.9E-03	2.72	up	1.3E-03
*AT5G08790*	*ANAC081*	2.57	up	2.6E-03	1.02	up	8.0E-01	4.59	up	7.6E-04
*AT5G14490*	*ANAC085*	4.50	up	3.1E-02	1.16	down	7.1E-01	5.40	up	1.5E-02
*AT5G22380*	*ANAC090*	10.66	up	5.5E-04	1.20	up	6.4E-01	18.10	up	5.7E-03
*AT5G63790*	*ANAC102*	2.94	up	1.4E-03	1.28	up	9.0E-03	4.31	up	9.1E-04
*AT4G25480*	*DREB1A*	3.78	up	2.0E-02	1.50	down	3.8E-01	7.05	up	1.0E-02
*AT4G25490*	*DREB1B*	60.64	up	6.8E-04	1.53	up	2.9E-01	122.36	up	1.5E-03
*AT4G25470*	*DREB1C*	13.90	up	1.2E-03	1.37	up	2.1E-01	22.07	up	1.7E-03
*AT5G51990*	*DREB1D*	31.82	up	7.0E-04	1.13	up	2.7E-01	89.04	up	7.7E-04
*AT5G05410*	*DREB2A*	24.85	up	5.3E-04	2.55	up	1.7E-02	18.67	up	1.2E-03
*AT3G11020*	*DREB2B*	18.47	up	1.1E-03	1.29	up	3.6E-01	24.59	up	1.2E-03
*AT1G02820*	*LEA*	2.17	up	7.9E-03	1.02	down	9.1E-01	3.87	up	8.7E-04
*AT2G03740*	*LEA*	2.39	up	3.0E-03	1.12	up	6.0E-01	2.82	up	4.9E-02
*AT2G18340*	*LEA*	6.70	up	5.0E-03	2.04	down	6.0E-03	34.83	up	8.4E-04
*AT3G17520*	*LEA*	59.12	up	3.2E-03	1.25	down	9.1E-01	250.54	up	3.1E-02
*AT4G36600*	*LEA*	2.37	up	2.6E-02	1.77	down	2.9E-01	10.45	up	9.8E-03
*AT1G32560*	*LEA*	3.31	up	4.7E-03	1.97	up	9.5E-02	4.42	up	1.4E-02
*AT5G06760*	*LEA*	63.26	up	1.9E-03	1.13	down	7.9E-01	161.77	up	7.7E-04
*AT1G52690*	*LEA*	96.62	up	3.8E-03	2.07	down	3.7E-01	276.76	up	1.3E-03
*AT3G15670*	*LEA*	16.70	up	3.5E-03	1.41	down	6.6E-01	115.29	up	3.6E-03
*AT3G53040*	*LEA*	4.16	up	2.2E-02	1.53	up	5.0E-01	6.53	up	1.7E-02
*AT1G52680*	*LEA*	9.71	up	2.0E-02	1.83	down	1.8E-01	18.63	up	8.0E-03

### CK deficiency affects the expression of known stress-related genes in response to high salinity

Because CKs mediate salt stress responses, it is also desirable to identify genes whose expression was altered by CK deficiency during salt stress. Therefore, comparison M-S/W-S was designed for this purpose (Figure 1A). We identified a total of 3276 genes that were significantly (q<0.05) differentially expressed by at least two-fold under salt stress, among which 2354 genes were upregulated and 922 were downregulated (Figure 1B, Tables S6A and S6B). A Venn diagram analysis indicated that 43.92% of the upregulated and 57.48% of the downregulated gene sets identified in comparison M-S/W-S overlapped with the corresponding gene sets from comparison M-C/W-C (Figure 5, comparisons M-C/W-C and M-S/W-S). On the other hand, another Venn diagram analysis of comparisons W-S/W-C and M-S/W-S revealed that only 12.83% and 21.48% of up- and downregulated gene sets of comparison M-S/W-S overlapped with up- and downregulated gene sets of comparison W-S/W-C, respectively (Figure 5, comparisons W-S/W-C and M-S/W-S). These results might suggest that CKs, rather than high salinity stress, altered the expression profile of salt-treated *ipt1,3,5,7* when compared with salt-treated WT in comparison M-S/W-S.

As for regulatory genes, among the major stress-related TFs, all of the salt-inducible *NAC* and *ZFHD* genes, such as *ANAC055*, *ANAC072* and *ZFHD1*, which confer stress tolerance when overexpressed (as previously discussed), were also induced in salt-treated *ipt1,3,5,7* when compared with salt-treated WT. Moreover, more salt-inducible *NAC* genes were identified in comparison M-S/W-S than comparison M-C/W-C (Table S7). Additionally, we found that the *DREB1B* and *DREB1D* were induced in comparison M-S/W-S but not in comparison M-C/W-C (Table S7). Expression of *DREB1B* is induced by ABA, salt and cold stresses, while *DREB1D* is induced by ABA, salt and drought stresses [64], [65]. Overexpression of *DREB1B* and *DREB1D* was shown to enhance salt tolerance of transgenic rice plants and drought tolerance of transgenic *Arabidopsis* plants, respectively [66], [67], suggesting that combinatory effects of various types of TFs may contribute to improved salt and drought tolerance of the CK-deficient *ipt1,3,5,7* plants. Furthermore, CK deficiency may also induce the SOS pathway in response to salt stress because the *SOS3* gene, which encodes an EF-hand Ca^2+^-binding protein that functions as a calcium sensor for salt tolerance, was upregulated 2.36 fold in comparison M-S/W-S (Table S7). Recently, Yang et al. (2009) reported that overexpression of *SOS3* resulted in enhanced salt tolerance of *Arabidopsis* transgenic plants [68]. In addition, we observed that the expression of *HKT1;1*, encoding the well-known high affinity K^+^/Na^+^ co-transporter that works in coordination with SOS proteins to control Na^+^ and K^+^ homeostasis, was significantly increased in the *ipt1,3,5,7* mutant under both normal and salt stress conditions (Figure 2). Consistent with our results, treatment of WT with exogenous CKs decreased transcript abundance of *HKT1;1* [69]. The increased transcript abundance of *HKT1;1* might also contribute to salt tolerance of CK-deficient plants because (i) expression of *HKT1;1* was also upregulated significantly in roots of the salt-tolerant *arr1,12* mutant, (ii) overexpression of *HKT1;1*, specifically in the mature root stele, enhanced salt tolerance in *Arabidopsis* transgenic plants and (iii) loss-of-function of *HKT1;1* rendered mutant plants hypersensitive to salt stress [69]–[71].

Regarding functional proteins, many genes encoding glyoxalase I proteins, XTRs, GTs, GHs and defensins were also upregulated in comparison M-S/W-S, as found in comparison M-C/W-C. The functions of these proteins have been discussed previously. Interestingly, 12 LEA protein encoding genes were found to be upregulated in comparison M-S/W-S, whereas only two genes (*At1g54890* and *XERO1/At3g50980*) were upregulated in comparison M-C/W-C. Ten of these 12 genes were also induced by salt stress (Table S7). Several lines of evidence have demonstrated that transgenic plants overexpressing LEA encoding genes display enhanced salt-tolerant phenotypes [72], [73], although their precise functional roles remain to be determined.

### Effect of CK deficiency and/or salt stress on CK-related genes

**Figure 6:**
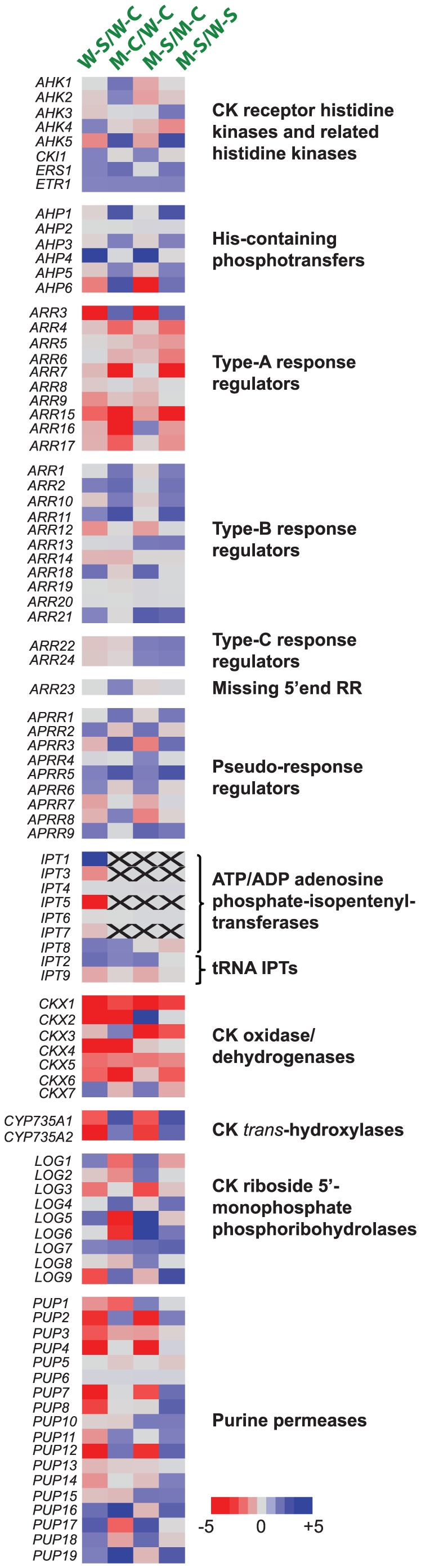
Heat map representation for the regulation of CK-related genes by CK deficiency under normal and salt stress conditions. Changes in expression levels are shown by color scales with saturation at five-fold changes. Blue and red color gradients indicate an increase or decrease in transcript abundance, respectively.

Because this study investigated the effect of CK deficiency on the expression of downstream genes under normal and salt stress conditions, it was desirable to examine the transcription of CK-related genes in detail. Therefore, we extracted the expression data for all of the genes that are known to be involved in CK metabolism (*IPT*, *CKX*, *CYP735A* and *LOG* genes), CK translocation (*PUP* genes) and CK signaling (*AHK*, *AHP*, *ARR* and *APRR* genes) [20], [74], [75]. The expression patterns of all of these genes were analyzed in all four comparisons and displayed in a heat map (Figure 6). We observed that the number of downregulation events (65) was more than three times that of upregulation events (21) (Table S8). In comparison W-S/W-C, among 92 genes analyzed, 25 genes were downregulated, of which a large portion included CK metabolism-related genes, while only three genes were upregulated, demonstrating a suppression of CK metabolism, which is consistent with a reduction in CK content under salt stress that was recently reported (Table S8) [16]. We also found that expression of the majority of *CKX* gene family members was decreased in all four comparisons (Figure 6, Table S8), which indicates that reduction of CK content caused by either salt stress or mutations in *ipt* genes diminishes expression of *CKX* genes. On the other hand, it has been reported that expression of *CKX* genes is induced by exogenous treatments of CKs [76], [77]. Taken together, these results suggest that the expression of *CKX* genes is CK-dependent.

In addition, among genes related to CK signaling, five genes were downregulated (one *AHP*, three type-A *ARR* and one type-B *ARR* genes), whereas only one gene, *AHP4*, was upregulated in the WT by salt treatment, indicating that CK signaling might be repressed by salt stress (Table S8, comparison W-S/W-C). These data suggest that a decrease in CK metabolism by salt stress will subsequently lead to repression of CK signaling. This provides a mechanism enabling plant adaptation to high salinity conditions. Indeed, it has been reported that suppression of CK signaling by mutations in genes encoding CK receptor kinases or type-B ARRs enhanced salt tolerance [18], [69]. It is worth mentioning that among 10 type-A *ARR* genes, more than half were downregulated in comparison M-C/W-C (five genes) and comparison M-S/W-S (six genes), but only one type-A *ARR* gene was downregulated in comparison M-S/M-C (Table S8), indicating that salt stress-responsive expression of type-A *ARR* genes is CK-dependent.

Among 18 *PUP* genes, which were reported to be involved in CK transport [20], [78], nine, two and four genes were downregulated in comparisons W-S/W-C, M-C/W-C and M-S/M-C, respectively, while none of the *PUP* genes had a diminished expression level in comparison M-S/W-S. At the same time, we detected a total of one, two, one and one upregulated genes in comparisons W-S/W-C, M-C/W-C, M-S/M-C and M-S/W-S, respectively (Figure 6, Table S8). Therefore, the transcription of the *PUP* genes depends more on salt stress than on CK levels. Brenner et al. (2005) reported that among 15 *PUP* genes analyzed, only expression of the *PUP4* gene was lower in WT plants treated with CKs, which further supports the CK-independent expression of the majority of the *PUP* gene family [59].

### Conclusions

When compared to WT under both normal and salt stress conditions, our genome-wide microarray analyses have detected transcriptional changes of more than 10% of all annotated *Arabidopsis* genes in the CK-deficient *ipt1,3,5,7* mutant. These genes encode proteins with a wide-range of functions, demonstrating the importance of CKs in the regulation of various biochemical, physiological and developmental processes. Downregulation of photosynthesis-related genes may cause shoot growth retardation, while upregulation of NAC TF encoding genes may play a role in enhanced lateral root development of the CK-deficient plants. The data gained through this study also suggest that CKs influence the established transcriptional networks, such as the *DREB*, *NAC* and *ZFHD* pathways, as well as ion signaling pathways, such as the SOS pathway. Ultimately, the impact of CKs on these regulatory networks results in optimized adaptation of sessile plants to adverse conditions. Overall, the results provided by this study may explain, at least in part, the phenotypic differences in plant growth and development between the WT and CK-deficient plants, as well as the enhanced tolerance to salt stress conferred by CK deficiency.

## Supporting Information

Table S1 **Primers used for qRT-PCR.**
(XLS)

Table S2 **Gene expression profiles of WT and the CK-deficient** ***ipt1,3,5,7*** **mutant under normal and salt stress conditions retrieved from microarray analyses.**
(RAR)

Table S3 **List of genes upregulated and downregulated in comparison W-S/W-C (WT-200 mM vs WT-0 mM NaCl).**
(XLS)

Table S4 **List of genes upregulated or downregulated in comparison M-C/W-C (** ***ipt1,3,5,7*** **-0 mM NaCl vs. WT-0 mM NaCl).**
(XLS)

Table S5 **List of genes upregulated or downregulated in comparison M-S/M-C (** ***ipt1,3,5,7*** **-200 mM NaCl vs.** ***ipt1,3,5,7*** **-0 mM NaCl).**
(XLS)

Table S6 **List of genes upregulated or downregulated in comparison M-S/W-S (** ***ipt1,3,5,7*** **-200 mM NaCl vs. WT-200 mM NaCl).**
(XLS)

Table S7 **Comparative analysis of upregulated genes found in comparison M-S/W-S (** ***ipt1,3,5,7*** **-200 mM NaCl vs. WT-200 mM NaCl).**
(XLS)

Table S8 **Comparison of changes in expression profiles of CK-related genes in WT and** ***ipt1,3,5,7*** **plants under normal and salt stress conditions.**
(XLS)
